# Secondary epiretinal membrane after Ex-Press glaucoma filtration device implant

**DOI:** 10.1007/s00417-020-05025-8

**Published:** 2020-12-01

**Authors:** Pasquale Loiudice, Francesco Sartini, Michele Figus, Marco Nardi, Chiara Posarelli, Giamberto Casini

**Affiliations:** Ophthalmology Unit, Department of Surgical, Medical, Molecular Pathology and of Critical Area, University of Pisa, Via Savi, 10, 56126 Pisa, Italy

**Keywords:** Cellophane macular reflex, Epiretinal membrane, Ex-press glaucoma filtration device, Glaucoma surgery, Macular pucker, Pre-macular fibrosis, Primary open-angle glaucoma

## Abstract

**Purpose:**

To estimate the frequency of epiretinal membrane (ERM) in eyes with primary open-angle glaucoma (POAG) treated with Ex-Press shunt implant. Secondarily, we aimed to assess the role of concomitant cataract surgery and the impact of the ERM on central foveal thickness and macular volume.

**Methods:**

In this prospective, consecutive, case-control study, we enrolled 54 patients affected by PAOG and scheduled for Ex-Press device implant with or without contemporary phacoemulsification. Contralateral eyes affected by POAG and receiving anti-glaucomatous eyedrops constituted the control group. Complete ophthalmologic evaluation and spectral-domain optical coherence tomography (OCT) were performed before and 1, 3 and 6 months after surgery.

**Results:**

Twenty-six eyes received the Ex-Press implant alone, and 28 eyes underwent the combined procedure. Six months postoperatively, we observed 18 (33%) cases of ERM: 15 (28%) of cellophane macular reflex (CMR) and 3 (6%) of pre-macular fibrosis (PMF). In the control group, 9 (17%) eyes developed an ERM: 8 (15%) were CMR, and 1 (2%) was PMF. The frequency of ERM statistically differs between treated and contralateral eyes (*P* = 0.032, *χ*^2^ test). The ERM frequency did not statically differ between eyes subjected to simple or combined surgery (*P* = 0.846, *χ*^2^ test). Mean central foveal thickness and mean macular volume did not significantly differ between groups.

**Conclusion:**

The Ex-Press glaucoma shunt may increase the risk of ERM onset regardless of the concomitant cataract surgery; however, most cases were cellophane macular reflex with limited functional and anatomical impact.

**Supplementary Information:**

The online version contains supplementary material available at 10.1007/s00417-020-05025-8.

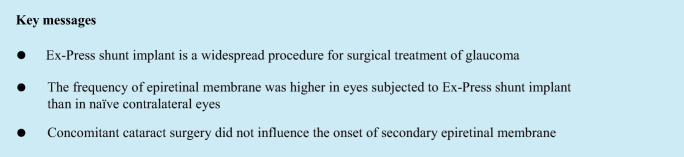



## Introduction

Epiretinal membrane (ERM) is a fibrocellular proliferation at the vitreomacular interface, associated with symptomatology ranging from entirely asymptomatic to severe visual impairment with metamorphopsia, photopsia, micropsia, or macropsia and decline of central vision [1]. The pathogenesis of ERM has not yet been fully elucidated although it is currently regarded as reactive gliosis [2–5].

Although ERM is idiopathic in most cases, several clinical conditions have been associated with the development or progression of secondary ERMs. These included retinal vascular disorders [6], diabetic retinopathy [7], inflammatory diseases, [8] blunt trauma [9], retinal vasoproliferative tumour [10], and ocular surgery [11]. Secondary ERM has been reported after cryotherapy and repeated intravitreal injections of bevacizumab for macular oedema in adult-onset Coats’ disease [12], following intravitreal injections for diabetic macular oedema [13] and after rhegmatogenous retinal detachment repair [14, 15].

It has been hypothesized that glaucoma filtration surgery may predispose one to the development and progression of ERM [16]. To date, trabeculectomy is considered the gold standard for the surgical treatment of glaucoma. It has been enhanced during years with the introduction of anti-metabolites, releasable, and adjustable sutures, and with creation and enlargement of sclerotomy using a scleral-trabecular punch instrument [17, 18]. However, the degree of filtration is still poorly predictable, and the procedure is still associated with sight-threatening complications, including hypotony maculopathy, choroidal detachment and effusions, choroidal haemorrhage, aqueous misdirection, hyphema, and cataract [19, 20]. For these reasons, various surgical devices have been developed to reduce adverse event frequency and severity, ensuring a significant IOP lowering at the same time.

The Ex-Press glaucoma filtration device (Alcon Laboratories, Fort Worth, TX, USA), approved by FDA in 2002, is a biocompatible, non-valved, stainless steel device and was designed as a variant of the classical trabeculectomy. Randomized prospective clinical trials compared the Ex-Press glaucoma filtration device implant with trabeculectomy in terms of safety, efficacy, postoperative visual recovery, and variance of IOP during the early postoperative period [21–23].

However, limited data are available in the literature regarding the association between the use of the Ex-Press glaucoma filtration device and disorders of the vitreoretinal interface. This study aimed to evaluate the frequency of ERM secondary to Ex-Press shunt implant in eyes affected by primary open-angle glaucoma. Secondarily, we aimed to assess the role of concomitant cataract surgery and the impact of the ERM on central foveal thickness and macular volume.

## Methods

In this prospective, consecutive, single-centre, case-control study, we included patients affected by primary open-angle glaucoma and scheduled for Ex-Press glaucoma filtration device implant with or without contemporary phacoemulsification. We received approval by the local Institutional Review Board (CEAVNO, Comitato Etico Area Vasta Nordovest, register number: 16554_CASINI). All the procedures were conducted in adherence to the tenets of the Declaration of Helsinki. Every patient signed an informed consent form.

Surgical procedures were performed by the same surgeon (M.N.) in the eye clinic of Pisa University Hospital (Pisa, Italy), between October 2018 and October 2019. All patients underwent a complete ophthalmic evaluation that included visual acuity assessment, Goldmann applanation tonometry, gonioscopy, standard automated perimetry, biomicroscopy, and optical coherence tomography (OCT) (Spectralis, Heidelberg Engineering, Heidelberg, Germany) at baseline and 1, 3, and 6 months postoperatively. The diagnosis of primary open-angle glaucoma was based on gonioscopy findings, optic disc appearance, and visual field defects on standard automated perimetry.

The inclusion criteria were diagnosis of primary open-angle glaucoma; indication for surgical treatment; contralateral eye affected by primary open-angle glaucoma; and receiving anti-glaucoma eyedrops. The exclusion criteria were previous or subsequent ocular laser or surgical treatments except for cataract surgery at least 24 months before enrolment, pre-existing retinal pathology (e.g. schisis, vascular retinal disorders. Epiretinal membrane, choroidal neovascularization, age-related macular degeneration), ocular trauma, and inflammatory disorders.

**Fig. 1:**
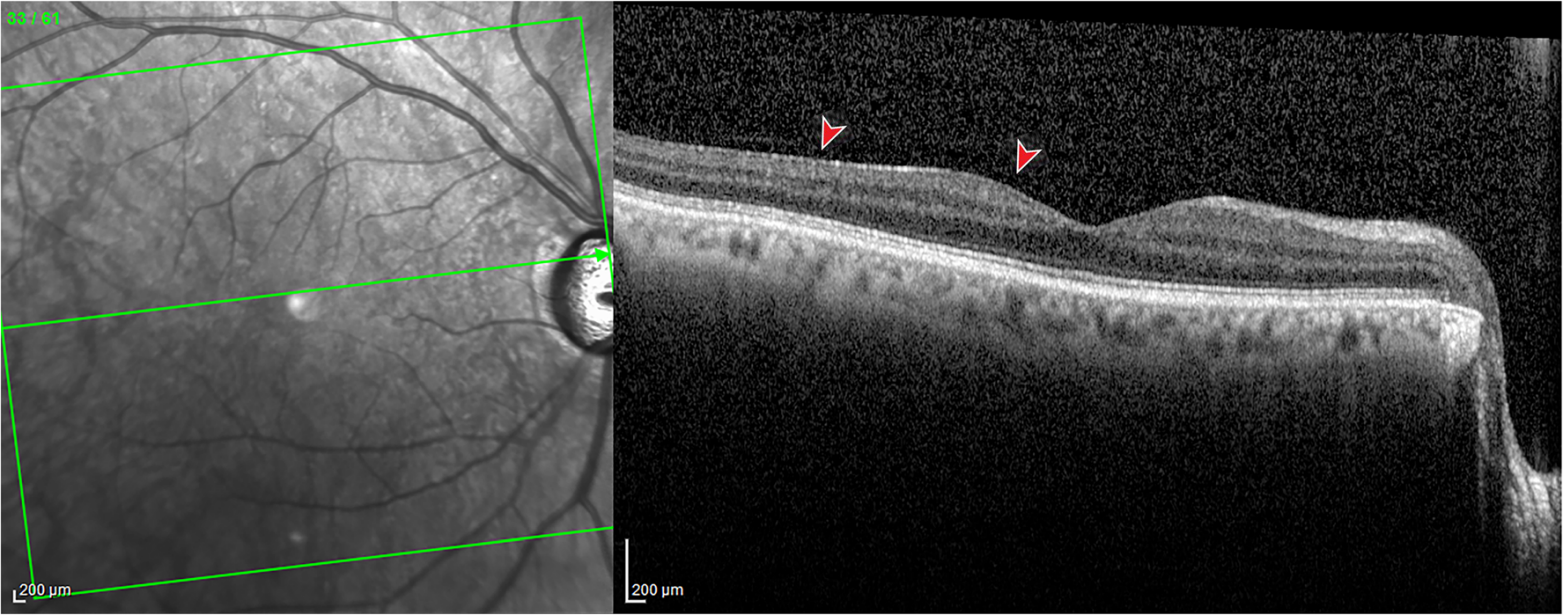
Spectral-domain optical coherence tomography of a patient with cellophane macular reflex. A hyperreflective layer (red arrowhead) is identifiable at the vitreomacular interface without foveal depression loss or alteration of the extrafoveal architecture

**Fig. 2:**
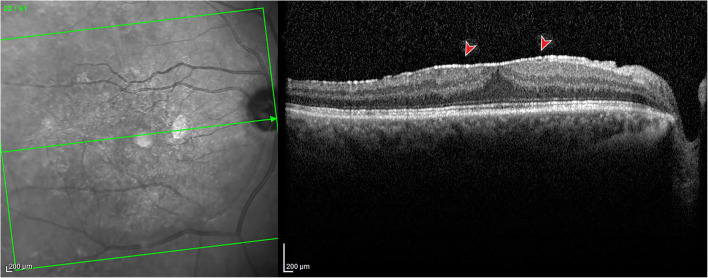
Spectral-domain optical coherence tomography of a patient with pre-macular fibrosis. A well-demarcated hyperreflective layer (red arrowhead) is identifiable at the vitreomacular interface, with foveal depression loss, increased central foveal thickness

ERM was categorized in two types: *cellophane macular reflex* (CMR) is defined as a hyperreflective layer at the vitreomacular interface without foveal depression loss or alteration of the extrafoveal architecture (Fig. 1). *Pre-macular fibrosis* (PMF) is defined as OCT hyperreflective layer with foveal depression loss, with or without intraretinal fluid, and alteration of the extrafoveal architecture due to the ERM contraction (Fig. 2) [24, 25].

OCT exams were evaluated by two blinded experienced observers. In case of discordance, the principal investigator reviewed and categorized. The occurrence of ERM was compared between treated eyes and control group (contralateral eyes affected by glaucoma and receiving anti-glaucoma eye drops) and between simple and combined glaucoma surgery. Central foveal thickness (CFT) and macular volume (MV) were calculated using the Early Treatment Diabetic Retinopathy Study (ETDRS) grid and were compared between groups. CFT was defined as the average thickness of the macula in the central 1-mm ETDRS grid. MV was defined as the sum of all volumes of all nine subfields.

### Surgical procedures

The Ex-Press P-200 glaucoma filtration device was implanted according to the standard procedure. Briefly, after limbal-based conjunctival dissection, a 3.0 mm × 3.0 mm partial-thickness scleral flap was obtained; mitomycin C 0.2 mg/ml was applied for 2 min and then thoroughly washed with balanced saline solution; a 25-gauge needle was used to make a microincision under the scleral flap in the blue-grey transition zone; the Ex-Press glaucoma device was inserted through the incision. Two releasable sutures and one everting suture were applied on the scleral flap according to the safe Ex-Press procedure [26, 27]; finally, the conjunctiva was closed. In case of combined surgery, standard two-port phacoemulsification using a clear cornea incision of 2.25 mm was performed after mitomycin C washout and before the microincision under the scleral flap. Corneal incisions were hydrated, and the corneal tunnel was closed with a 10-0 nylon suture.

### Statistical analysis

Statistical analysis was performed using SPSS Statistics version 25 (IBM Corporation, Armonk, NY, USA). Descriptive statistic was used to summarize mean values and standard deviations of all numerical data. Sample size was calculated using the effect size from the results of a previous similar study [16] and indicated that 54 subjects were required to detect a 25.7% difference in the incidence of ERM, with a power of 80% and a significance level of 0.05. Post-hoc analysis indicated that this study had a power of 90% with an actual α of 0.02 to detect a 24% difference in the incidence of ERM between treated eyes and controls. Distribution of values was assessed using Kolmogorov-Smirnov test and Shapiro-Wilk test. For normally and not normally distributed continuous variables, we used paired *t* test and Wilcoxon signed-rank test, respectively, to compare baseline with follow-up measures and independent *t* test and Mann-Whitney *U* test to compare variables between groups, respectively. χ^2^ test was used for categorical variables. *P* values < 0.05 were considered significant.

## Results

One hundred seventy-eight eyes of 89 consecutive patients, 51 males and 38 females, were eligible for this study. All patients had primary open-angle glaucoma and were scheduled for single or combined treatment. Thirty five patients were excluded from the study: 29 required further surgical procedure during the follow-up period (bleb needle revision or complete surgical revision), and 6 were lost to follow-up. The remaining 54 patients were included for the statistical analysis. Twenty eight eyes underwent phacoemulsification and Ex-Press glaucoma device implant, and 26 eyes received Ex-Press glaucoma device implant alone. Fifty four contralateral eyes, affected by primary open-angle glaucoma and receiving anti-glaucoma eyedrops, constituted the control group. Neither of the eyes had a history of laser or surgical treatment except for cataract surgery at least 24 months before enrolment. Demographic and preoperative features are summarized in Table 1.

**Table 1:** Secondary epiretinal membrane after Ex-Press glaucoma filtration device implant

Features	Number
Gender	
Male (%)	30 (56)
Female (%)	24 (44)
Eye	
Right (%)	22 (41)
Left (%)	32 (59)
Treatment	
Ex-Press implant (%)	26 (48)
Ex-Press implant + phacoemulsification (%)	28 (52)
Mean age ± SD (y)	71.08 ± 9.38
Mean IOP ± SD (mm Hg)	23.4 ± 3.8
Mean MD	− 17.18 ± 5.07
Mean PSD	8.66 ± 3.09
Mean number of eyedrops	2.75 ± 0.99

*IOP* intraocular pressure; *MD* mean deviation; *PSD* pattern standard deviation; *SD* standard deviation; *y* years

Demographic and preoperative features

Mean IOP of the study eyes significantly decreased from 29.9 ± 8.6 mmHg to 11.0 ± 3.1 mmHg at 6-month follow-up (*P* < 0.001, paired *t* test). No significant difference was observed in the control group between baseline and follow-up measurements (12.7 ± 1.9 mmHg and 12.8 ± 2.4 mmHg, respectively, *P* = 0.777, paired *t* test). The mean number of anti-glaucomatous eyedrops of treated eyes decreased from 2.75 ± 0.99 to 0.13 ± 0.44 postoperatively (*P* < 0.001, Wilcoxon signed-rank test). Mean deviation (MD) and pattern standard deviation (PSD) were collected from all patients. No difference was observed between baseline and postoperative values of mean MD (− 16.44 ± 4.68 and − 16.39 ± 4.19 respectively, *P* = 0.861, Wilcoxon signed-rank test) and mean PSD (8.41 ± 2.8 and 8.13 ± 2.8 respectively, *P* = 0.433, Wilcoxon signed-rank test) of treated eyes. Mean MD (− 11.25 ± 5.9 and − 11.56 ± 6.2, respectively) and mean PSD (6.69 ± 3.3 and 7.02 ± 3.3, respectively) of the control eyes did not significantly change between baseline at 6-month follow-up (*P* = 0.191 and *P* = 0.271, respectively, Wilcoxon signed-rank test).

Visual acuity significantly increased postoperatively in eyes subjected to combined surgery, from 0.21 ± 0.10 logarithm of minimum angle of resolution (logMAR) to 0.07 ± 0.08 logMAR (*P* < 0.001, paired *t* test). Visual acuity did not significantly change in the eyes not subjected to cataract surgery (from 0.162 ± 0.28 logMAR to 0.160 ± 0.26 logMAR, respectively, *P* = 0.929, paired *t* test).

Six months after surgery, we observed 18 cases (33%) of ERM: 15 (28%) of CMR and 3 (5%) of PMF. In the control group, 9 eyes (17%) developed an ERM: 8 (15%) were CMR and 1 (2%) was PMF. The frequency of ERM statistically differs between treated and contralateral eyes (*P* = 0.032, *χ*^2^ tests). The ERM frequency did not statically differ between eyes subjected to combined surgery (9 eyes, 32%) and eyes who were treated with the Ex-Press glaucoma filtration device alone (9 eyes, 35%) (*P* = 0.846, χ2 tests) (Table 2). Mean postoperative IOP (1 day after surgery) did not significantly differ between eyes who developed ERM and eyes who did not (15.25 ± 5.11 mmHg and 14.71 ± 7.27 mmHg, respectively, *P* = 0.752, unpaired *t* test).

**Table 2:** Secondary epiretinal membrane after Ex-Press glaucoma filtration device implant

	Treated				Controls	
	Ex-Press (*n* = 26)	PhacoEx-Press (*n* = 28)	Total (*n* = 54)	*P* ^a^	(*n* = 54)	*P* ^b^
Epiretinal membrane				0.846		0.032
CMR (%)	8 (31)	7 (25)	15 (28)		8 (15)	
PMF (%)	1 (4)	2 (7)	3 (6)		1 (2)	

*CMR* cellophane macular reflex, *PMF* paramacular fibrosis

^a^Single versus combined surgery (χ^2^ test); ^b^treated versus controls (χ^2^ test)

Frequency of epiretinal membrane at 6 months after surgery

CFT and MV were compared between treated and control eyes at baseline and 6-month follow-up visit. No significant difference was observed at baseline between the groups (*P* = 0.542 and *P* = 0.682, respectively, *t* test). Postoperatively, mean CFT of treated eyes was 265.59 ± 32.30 μm, and mean CFT of controls was 261.52 ± 31.86; the difference was not significant (*P* = 0.511, *t* test). Mean MV did not significantly differ between treated and contralateral eyes (7.78 ± 0.45 mm^3^ and 7.86 ± 0.41 mm^3^, respectively, *P* = 0.356, *t* test). In a subgroup analysis, we compared CFT and MV between subjects with and without the ERM within treated eyes. The difference was not statistically significant (270.56 ± 27.02 μm and 263.11 ± 34.73 μm, respectively, *P* = 0.392; 7.80 ± 0.45 mm^3^ and 7.77 ± 0.46 mm^3^, respectively, *P* = 0.763, *t* test). No difference in mean CFT and MV is observed within treated eyes between simple and combined surgery (262.65 ± 31.48 μm and 268.32 ± 33.38 μm, respectively, *P* = 0.525; 7.65 ± 0.45 mm^3^ and 7.90 ± 0.43 mm^3^, respectively, *P* = 0.057, *t* test) (Table 3).

**Table 3:** Secondary epiretinal membrane after Ex-Press glaucoma filtration device implant

	Treated						Controls	
	Ex-Press	Phaco Ex-Press	*P* ^a^	ERM	No ERM	*P* ^b^		*P* ^c^
CFT (mean ± SD)	261.71 ± 32.10	269.42 ± 35.12	0.423	274.69 ± 33.92	261.50 ± 33.09	0.198	261.36 ± 33.13	0.515
MV (mean ± SD)	7.64 ± 0.43	7.90 ± 0.48	0.057	7.87 ± 0.54	7.73 ± 0.43	0.347	7.86 ± 0.43	0.192

*CFT* central foveal thickness; *MV* macular volume; *ERM* epiretinal membrane

^a^Single versus combined surgery (*t* test); ^b^eyes with ERM versus eyes without ERM (*t* test)

^c^Treated versus controls (*t* test)

Central macular thickness and central macular volume at 6 months after surgery

## Discussion

The prevalence of idiopathic ERM has been estimated by several population-based epidemiology studies and ranges from 2.2 to 26.1% [25, 28, 29]. The discrepancy in the prevalence of ERM among studies may be determined by the different populations considered and the method used to detect the ERM (retinography, OCT, or both). Studies [24, 30] proved that the presence of ERM (CMR or PMF) was better detected by OCT rather than biomicroscopy or fundus photography. It has been observed that the number of cases of ERM increases with age, with a peak between 70 and 79 years [25]. Bilateral forms have been reported in 19.5–31% of patients [25]. Several risk factors have been identified, including age, female gender, ethnicity, cataract surgery, retinal vein occlusion, diabetes, myopia, and hypercholesterolemia [11, 31, 32].

Secondary epiretinal membrane has been observed after cataract surgery with an incidence of 16.8%, [25] and following pars plana vitrectomy for rhegmatogenous retinal detachment with an incidence ranging from 4.4 to 12.8% [33, 34].

The ERM incidence after glaucoma surgery has been investigated by Vieria et al. in 2016 [16]. In their retrospective study, they analysed the development and progression of ERM after trabeculectomy for primary open-angle glaucoma. PMF was observed in 9/50 eyes (18%) and CMR in 19/50 eyes (38%). Of the 16 eyes that had a preoperative OCT, 3 (18.8%) developed ERM, and 4 (25%) progressed from CMR to PMF. No significant difference in ERM frequency was found comparing patients who underwent trabeculectomy alone or combined with phacoemulsification.

The frequency of the ERM in our study after Express glaucoma device implant was 33% and was lower than the reported one after trabeculectomy (56%) [16]. Interestingly, in most of our cases (83%), the ERM was a CMR and did not affect macular profile or symptomatology. In the two cases of PMF, best corrected visual acuity did not significantly differ from preoperative values, and patients did not complain of blurred, distorted vision or any other symptoms, and did not require surgical intervention in the follow-up period of this study.

The frequency of ERM after glaucoma surgery was higher than that reported after cataract surgery. It has been hypothesized that the longer operative time and the entity of the forces acting on vitreoretinal interface may contribute to explain this observation [16]. Giambruni et al. did not demonstrate an association between the use of prostaglandin analogues and the development of ERM [35].

The pathophysiology of ERM development after Ex-Press shunt surgery remains largely unknown. Hypotony following Ex-Press shunt surgery may be a contributing factor to the subsequent development of ERM. To evaluate the effect of postoperative hypotony in this study population, we compared postoperative IOP between subjects who developed ERM and patients that did not develop ERM. We did not find any significant difference in early postoperative IOP between the 2 subgroups.

The Ex-Press shunt implant procedure does not typically require an iridectomy and involves less tissue removal than conventional trabeculectomy surgery. Made of stainless steel, histological biocompatibility animal models have demonstrated minimal inflammatory and scarring reactions after Ex-Press shunt implant in rabbits [36]. The increased levels of postoperative inflammation and the rapid variation of intraocular pressure that occur after trabeculectomy may provoke shear stress on the retinal surface. This may induce or accelerate a posterior vitreous detachment and trigger a gliotic response.

The surgical technique may also have a role. In our case, all surgeries were performed according to the safe Ex-Press procedure in which two releasable sutures and one everting suture were applied on the scleral flap. The knots were initially tight; as IOP increased in the postoperative period, the releasable sutures were removed. Finally, pulling on the everting suture, a lift of the scleral flap was obtained. The technique ensures good control of filtration avoiding rapid variation of the IOP and early postoperative hypotony.

The role of cataract surgery was analysed comparing the outcomes of eyes subjected to simple or combined surgery. We found that phacoemulsification did not significantly influence the frequency of ERM during Ex-Press glaucoma surgery, and this was in accordance with the research of Vieria and colleagues [16].

In the current study, 17% of contralateral eyes developed an ERM in the considered postoperative period. This finding is in accordance with the data from previous epidemiological studies (2.2–26.1%) [25, 29]. It is important to note that the mean age of our patients (70.3 ± 7.8 years) coincides with the peak of incidence of ERM reported by the Blue Mountains Eye Study [25].

The mean postoperative values of CFT and MV observed in our research were comparable to the reported average measures in healthy individuals, ranging from 255.4 to 271.4 μm and from 6.76 to 8.53 mm^3^, respectively [37]. Mean CFT and mean MV did not significantly change between treated eyes and controls and between eyes subjected to simple or combined surgery. Analysing mean CFT and mean MV of the eyes who developed an ERM, no significant difference was observed if compared to treated eyes with no ERM at follow-up. This is in accordance with the observation that most cases had a CMR that did not affect the foveal profile. Furthermore, OCT has been considered more sensitive to detect early cases of ERM that could be overlooked by biomicroscopy or fundus retinography.

A limitation of this study is the subjective evaluation of the OCT images. However, there was an agreement between the masked observers in all cases. Each of the examiners analysed the raw images (every single B-scan) of the macular area to identify any artefact. In the current study, we excluded all cases in which further surgical procedures were performed to avoid potential confounding factors.

In conclusion, the Ex-Press glaucoma filtration device implant may increase the risk of ERM onset regardless of the concomitant cataract surgery. However, most cases were CMR, with limited clinical implications in terms of best-corrected visual acuity impairment, distorted vision, or macular profile alteration.

## Supplementary Information

ESM 1 (XLSX 18 kb)

## Data Availability

Data are available on request.
